# The Chemical Profile, Antioxidant, and Anti-Lipid Droplet Activity of Fluid Extracts from Romanian Cultivars of Haskap Berries, Bitter Cherries, and Red Grape Pomace for the Management of Liver Steatosis

**DOI:** 10.3390/ijms242316849

**Published:** 2023-11-28

**Authors:** Oana Craciunescu, Ana-Maria Seciu-Grama, Elena Mihai, Elena Utoiu, Ticuta Negreanu-Pirjol, Carmen Elena Lupu, Victoria Artem, Aurora Ranca, Bogdan-Stefan Negreanu-Pirjol

**Affiliations:** 1 National Institute of R&D for Biological Sciences, 060031 Bucharest, Romania; anamaria.seciu@incdsb.ro (A.-M.S.-G.); elena.mihai@incdsb.ro (E.M.); elena.utoiu@incdsb.ro (E.U.); 2 Faculty of Pharmacy, University Ovidius of Constanta, 900470 Constanta, Romania; clupu@univ-ovidius.ro (C.E.L.); bogdan.negreanu@univ-ovidius.ro (B.-S.N.-P.); 3 Academy of Romanian Scientists, Ilfov Street, No. 3, 050044 Bucharest, Romania; 4 Research-Development Station for Viticulture and Winemaking of Murfatlar, 905100 Murfatlar, Romania; artemv@statiuneamurfatlar.ro (V.A.); director@statiuneamurfatlar.ro (A.R.)

**Keywords:** *Lonicera caerulea*, *Prunus avium* syn. *Cerasus avium*, *Vitis vinifera*, composition, phenolic compounds, steatosis, fatty acids, antioxidant, lipid peroxidation

## Abstract

This study aimed to investigate, for the first time, the chemical composition and antioxidant activity of fluid extracts obtained from three Romanian cultivars of haskap berries (*Lonicera caerulea* L.) var. Loni, bitter cherries (*Prunus avium* var. *sylvestris* Ser.) var. Silva, and pomace from red grapes (*Vitis vinifera* L.) var. Mamaia, and their capacity to modulate in vitro steatosis, in view of developing novel anti-obesity products. Total phenolic, flavonoid, anthocyanin, and ascorbic acid content of fluid extracts was spectrophotometrically assessed and their free radical scavenging capacity was evaluated using Trolox Equivalent Antioxidant Capacity (TEAC) and free 2,2-diphenyl-1-picrylhydrazyl (DPPH) radical inhibition assays. The Pearson coefficients showed a moderate correlation between the antioxidant activity of fluid extracts and their phenolic content, but a strong correlation between anthocyanin and ascorbic acid content. HPLC analysis identified and quantified the main phenolic compounds of chlorogenic and syringic acid, catechin, and glycosylated kaempferol, apigenin, and quercetin, in variable proportions. An in vitro experimental model of steatosis was developed in HepG2 hepatocytes treated with a mixture of free fatty acids. Cell culture analyses showed that cytocompatible concentrations of fluid extracts could significantly reduce the lipid accumulation and inhibit the reactive oxygen species, malondialdehyde, and nitric oxide secretion in stressed hepatocytes. In conclusion, these results put an emphasis on the chemical compounds’ high antioxidant and liver protection capacity of unstudied fluid extracts obtained from Romanian cultivars of bitter cherries var. Silva and pomace of red grapes var. Mamaia, similar to the fluid extract of haskap berries var. Loni, in particular, the positive modulation of fat deposition next to oxidative stress and the lipid peroxidation process triggered by fatty acids in HepG2 hepatocytes. Consequently, this study indicated that these fluid extracts could be further exploited as hepatoprotective agents in liver steatosis, which provides a basis for the further development of novel extract mixtures with synergistic activity as anti-obesity products.

## 1. Introduction

Fatty liver disease or liver steatosis is a chronic inflammatory disease that refers to an increase of up to 5–10% intracellular fat reported in the liver’s weight due to lipid accumulation from a high-fat diet, and affects ~30% of the general population, in particular from industrialized countries [1]. The metabolism is thus disturbed through defective fatty acid oxidation, leading to an increased production of reactive oxygen species (ROS) and redox and lipid peroxidation chain reactions [2]. The triggering of major signaling pathways, such as nuclear factor kappa-B (NF-kB) and c-Jun N-terminal kinase (JNK), results in the release of proinflammatory cytokines and low grade inflammation [3]. Thus, in order to control the processes involved in non-alcoholic fatty liver disease (NAFLD), novel agents with antioxidants and a capacity to inhibit fat accumulation and lipid peroxidation reaction cascades are currently being researched.

Haskap, named blue honeysuckle or honeyberry (*Lonicera caerulea* L.), is a shrub with edible dark blue fruits, commonly known as *L. caerulea* var. *kamtschatica* Sevast. and *L. caerulea* var. *edulis* in cold climates from the northern hemisphere regions of Asia, Europe, and North America, and presenting two endemic varieties of *L. kamtschatica* var. *villosa* in Canada and *L. kamtschatica* var. *emphyllocalyx* in Japan [4,5]. The fruit has a high content of ascorbic acid and anthocyanins, and various types of extract showed correlated antioxidant activity [6,7]. In addition, anti-inflammatory, antiproliferative, and antidiabetic activity were previously proved in vitro and in vivo, together with other health benefits, including the protection of the cardiovascular and nervous systems [8,9,10]. The antioxidant and anti-inflammatory activity of haskap berry extract was demonstrated in fibroblast-like BRL 3A cells isolated from rat liver through the inhibition of several proteic markers and mRNAs expression, together with the suppression of caspases in the lipopolysaccharide (LPS)-induced apoptosis of cells [11]. Moreover, the activity of the antioxidant enzymes superoxide dismutase and catalase increased in HepG2 cells treated with 300 μg/mL haskap berry extracts [12]. It was also reported that the Canadian haskap berry extract with the highest phenolics content could control cyclooxygenase-2 activity in a similar proportion as diclofenac in LPS-inflamed THP-1-derived macrophages [13]. A 25% ethanolic extract of Korean haskap berries could regulate the AMP-activated protein kinase (AMPK) and acetyl-CoA carboxylase (ACC) signaling pathways in HepG2 cells in diet-induced obese mice, thus reducing lipid accumulation and ameliorating NAFLD [14]. In addition, the suppression of lipogenesis in murine adipocytes was correlated to the phenolics content of Chinese haskap berry extract in 70% ethanol [15]. Extracts from other haskap parts, like leaves, branches, and flowers, were indicated as active agents for the prevention and treatment of type II diabetes [16], inflammation-related diseases [17], and experimental murine colitis [18], respectively. No studies on the chemical composition of Romanian selections of *L. caerulea* var. *edulis* berry extracts and their phytotherapeutic properties were found.

Bitter foods are traditionally known to help digestion and nutrient absorption. The wild cherry or bird cherry (*Prunus avium* syn. *Cerasus avium* var. *sylvestris* Ser.) originates from Europe, Asia Minor, Western Siberia, and Northern Africa. Several Romanian genotypes of wild cherry were characterized by an average-to-high intensity of bitter taste [19], high polyphenolic content, and good processing properties [20]. Previous studies have indicated that 80% ethanol Egyptian black cherry (*P. serotina* Ehrh.) extract had liver protective action in rats exposed to the toxicity of acetaminophen by decreasing malondialdehyde (MDA) content and increasing catalase activity [21], while the antiproliferative activity of 70% ethanolic extract of Portuguese cultivar of sweet cheery fruit (*P. avium* L. var. Saco) was reported in hepatocarcinoma cells [22]. Also, 96% ethanol pulp extract combined with degreased grounded seeds of sour cherry (*P. cerasus* L.) could prevent liver steatosis in diet-induced obesity in rats by decreasing the serum level of glycaemia, triglycerides, and thiobarbituric reactive substances (TBARSs) [23]. Other studies on the fruits and leaves of different varieties of sweet and sour cherries showed that the bioactive compounds exhibited a high potential to inhibit the chemical substrates of enzymes related to diabetes [24,25]. Other by-products of Portuguese sweet cherry (*P. avium* L.), i.e., flowers, leaves, and stems, were good sources of phenolic extracts with anti-inflammatory activity found in murine RAW 264.7 macrophages, and presented antimicrobial potential against Gram-positive bacterial strains [26], with antidiabetic and anti-hemolytic potential [27]. No studies on the effect of bitter cherry extracts on NAFLD were found.

Pomace obtained from red grapes (*Vitis vinifera* L.) during winemaking consists of skin, pulp, stalk, and seed residues and is rich in phenolics with health-beneficial properties exerted through lipid oxidation inhibition and antimicrobial activity and can be useful in food fortification [28,29]. However, its direct effect on liver protection against lipid accumulation was not yet reported. Grape pomace extract of the Merlot variety revealed antiproliferative and bacteriostatic potential, but the effect decreased after in vitro simulated digestion and colonic fermentation [30]. Previous studies have indicated a high anti-inflammatory effect of red grape pomace treated with tannase in interleukin-1β (IL-1β)-inflamed Caco-2 intestinal cells through the inhibition of prostaglandin E2 and proinflammatory cytokine IL-8 due to an increase in aglycone compound content [31]. The in vivo protective activity of various extracts of the fruits of *V. vinifera* varieties from different geographical areas [32,33], and the by-products of *V. vinifera* root [34], leaves [35], and stem bark [36], and grape seeds and oil [37,38,39,40], was previously reported in toxic liver disease. A polysaccharidic extract of Chinese Cabernet Sauvignon grape pomace showed the biochemical and histological recovery of CCl_4_-caused liver injury in mice [41]. No studies on the in vitro effect of the ethanolic extract of red grape pomace, rich in phenolic compounds, were found on NAFLD.

The chemical composition of fruit extracts varies according to cultivar, geographical area, season, and extraction method, and their biological properties are influenced by storage conditions and extraction parameters. The literature showed the variable composition and pleiotropic activity of the studied species extracts, but it was not exhaustive. Currently, there are no standardized extracts or precise dosages to develop nutra-/pharmaceutical products for fat liver protection and anti-obesity.

In this context, the aim of the present study was to investigate, for the first time, the chemical composition and in vitro hepatoprotective effect of the fluid extracts of Romanian cultivars of haskap berries (*L. caerulea* L.) var. Loni, along with that of bitter cherries (*P. avium* var. *sylvestris* Ser.) var. Silva and pomace obtained from red grapes (*V. vinifera* L.) var. Mamaia, and in particular, their action against fat deposition, oxidative stress, and lipid peroxidation processes triggered by fatty acids in HepG2 hepatocytes, in order to advance in development of novel products for the prevention and treatment of NAFLD.

## 2. Results and Discussion

### 2.1. Composition of Fluid Extracts

The fluid extracts were prepared using a simple technology based on ultrasonication, which can be easily scaled up. The extraction yield was 12.94% for haskap, 4.62% for bitter cherry, and 9.32% for red grape pomace. The total phenolic, flavonoid, anthocyanin, and ascorbic acid content in the fluid extracts of haskap, bitter cherry, and red grape pomace Mamaia cultivars is presented in Table 1.

The registered data showed that grape pomace fluid extract had higher total phenolic and flavonoid content than haskap and the lowest value was found in bitter cherry extract. Anthocyanins and ascorbic acid content was also found in high quantities, in particular in haskap and grape pomace fluid extracts. It is known that the great variability of phytochemical properties is due to cultivation conditions and varieties of cultivars. However, it is worth mentioning some comparable values. Previous studies showed a range of 2.5–52 mg/g d.w. for total phenolic content in *L. caerulea* varieties harvested from different Lithuanian periods of ripening [42], and a value of 260 mg/g phenolic content in the Italian red grape pomace [43], while *P. avium* varieties from Spain had values between 0.86 and 2.84 mg/g [44]. Regarding ascorbic acid, it was previously highlighted that haskap fruits had the highest content among other berries [4].

The main phenolics present in the fluid extracts were identified via HPLC, according to the used standards, and quantitative results are provided in Table 2. Thus, the identified phenolic compounds were chlorogenic acid, catechin hydrate, syringic acid, and glycosylated flavonoids in haskap berry extract, while catechin hydrate and glycoside derivatives of quercetin and kaempferol were found in pomace fluid extract. Bitter cherry extract presented significant quantities of chlorogenic, caffeic and syringic acids, apigenin, and kaempferol glycosides.

Similar studies have identified catechin, quercetin, and rutin as the most important phenolics in the fresh berries of Polish haskap (*L. kamtschatica*) [45], while catechin, quercetin, kaempferol, and syringic and vanillic acid were representative of extracts of pomace from Chilean red grape varieties [46]. A recent study revealed phytochemicals like quercetin, syringic, ferulic, and gallic acid in the ultrasound-assisted extract of Pakistan sweet cherry (*P. avium*) [47]. In addition, several authors reported that the most abundant anthocyanin compound was cyanidin-3-glucoside ranging between 79 and 92% in haskap berries [4] and cyanidin-3-rutinoside reaching 95% in red sweet cherries [48], while malvidin-3-glucoside represented 70% in Mediterranean grape pomace [49].

NAFLD is a lipid metabolic dysfunction that leads to obesity and it is associated with diabetes through insulin resistance and cardiovascular disease through hypertension and atherosclerosis. Molecular mechanisms driven by fatty-acid-binding protein (FABP-1), fatty acid transport proteins (FATPs), and the cluster of differentiation 36 (CD 36) markers are involved in fatty acid uptake; three enzymes of ACC, fatty acid synthase (FAS), and stearoyl-CoA desaturase-1 (SCD1) regulate lipogenesis; and carnitine palmitoyltransferase 1 (CPT1) regulated by peroxisome proliferator-activated receptor-α (PPARα) is the pathway activated for targeting the mitochondrial, peroxisomal, and cytochrome oxidation of fatty acids in NAFLD [50]. The alteration of these signaling pathways can lead from fat accumulation to inflammation and fibrosis of the liver. Currently, there are no drugs approved for NAFLD, but natural antioxidants, such as flavonoids, can be useful in limiting ROS production, lipid peroxidation, and stimulating fat burn. In this regard, it was reported that the action of quercetin on adipogenic factor C/EBPα gene expression to block adipogenesis, and on FAS and ACC gene levels to decrease lipogenesis, ultimately reduced total body fat percentage [51,52]. Catechins were also responsible for AMPK, which reduced fat deposition in liver and increased fatty acid oxidation [53]. Syringic and chlorogenic acid, also found in the composition of fluid extracts from the present study, acted as natural anti-steatotic agents through regulation of lipid metabolism, reduced the level of plasma lipids, and altered the expression of lipogenesis genes in obese mice [54,55].

### 2.2. Free Radical Scavenging Capacity

The correlation of the chemical composition and antioxidant activity of fluid extracts was further investigated. TEAC and free DPPH radical inhibition values are presented in Table 3. The data showed that TEAC values were similar for *L. caerulea* and grape pomace fluid extracts and slightly higher than the antioxidant capacity of *P. avium* extract. Moreover, TEAC values of haskap and grape pomace fluid extracts were higher than 1 mM TE, indicating a great capacity for free radical scavenging, as reported with Trolox, the synthetic analogue of vitamin E, known as an antioxidant agent. Likewise, free DPPH radical inhibition took place in a significantly higher proportion in the presence of *L. caerulea* and grape pomace extracts than that of *P. avium* extract.

It should be emphasized that the antioxidant activity of haskap fluid extract was close to that of pomace extract from red grapes var. Mamaia, although its total phenolic content was only one third of that of pomace extract. The calculated Pearson correlation coefficients were between 0.6575 and 0.7453, indicating the moderate correlation of TEAC and DPPH inhibition values to the content of total phenolics and flavonoids in the analyzed fluid extracts (Table 4). This could be due to the presence of other types of bioactive compounds also exerting antioxidant activity, from which ascorbic acid played a significant role. We observed a strong correlation of free radical scavenging capacity of the fluid extracts to total anthocyanin content (r = 0.9211–0.9579) and also between TEAC values and ascorbic acid content (r = 0.8142).

The high antioxidant activity of dark-colored berries and red grape pomace extracts is mainly the result of their composition that is rich in phenolic compounds, which has a characteristic structure with several hydroxyl groups. The redox properties of these compounds are exerted through both hydrogen atom transfer (HAT) and single electron transfer (SET) mechanisms of action [56]. Nevertheless, recent HPLC coupled to mass spectrometry and factorial analysis demonstrated synergistic free radical scavenging activity of the majority of tested polyphenols, such as quercetin-4′-glucoside, catechin, epicatechin, and caffeoyl and coumaroylquinic acid derivatives [57]. Still, the study showed that procyanidin B1 and chlorogenic acids presented only independent action. Taking into account that all three extracts analyzed in the present study had a high content of anthocyanins and chlorogenic acid, this could be another reason for the moderate correlation between the antioxidant activity of the fluid extracts of haskap, bitter cherry, and pomace of red grapes and their total phenolic content.

### 2.3. Cell Culture Testing of Liver Steatosis Modulation

#### 2.3.1. Cytocompatibility of Fluid Extracts in HepG2 Hepatocytes

**Figure 1:**
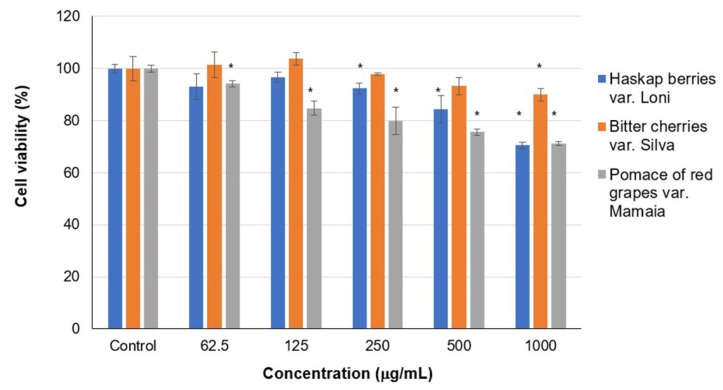
Cell viability of HepG2 cultivated in the presence of different concentrations of fluid extracts of haskap berries var. Loni, bitter cherries var. Silva, and pomace of red grapes var. Mamaia. The results are expressed as mean ± SD (*n* = 9); * *p* < 0.05, compared to control.

The results of cell viability in HepG2 cultivated in the presence of fluid extracts are presented in Figure 1.

The data showed that all fluid extracts were cytocompatible (cell viability > 80%) in HepG2 culture, in a variable range of concentrations. Thus, haskap extract was cytocompatible up to 500 μg/mL, bitter cherry extract up 1000 μg/mL, and grape pomace extract up to 250 μg/mL. For these concentrations, the cell viability varied between 80 and 103%. Higher concentrations of haskap and grape pomace extracts induced a decrease in cell viability down to 70%, indicating slight cytotoxicity. These observations indicated the selection of fluid extract concentrations of 62.5, 125, and 250 μg/mL, as well as cytocompatible in HepG2 cell culture, and were used for further testing.

#### 2.3.2. Hepatoprotective Effect in FFA Steatosis Model

**Figure 2:**
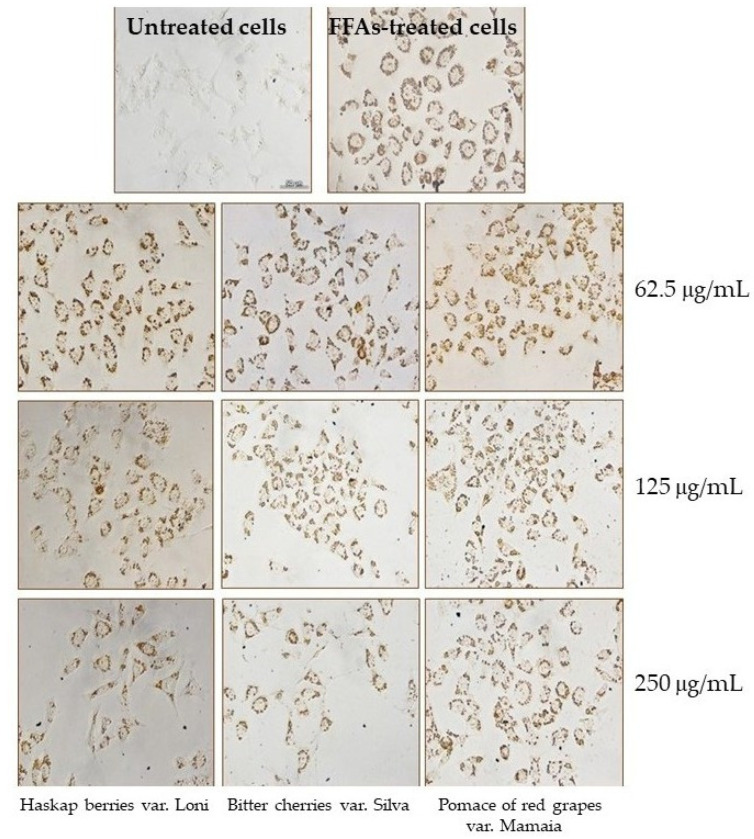
Micrographs of HepG2 cells treated with FFAs in the presence of different concentrations of fluid extracts of haskap berries var. Loni, bitter cherries var. Silva, and pomace of red grapes var. Mamaia, presenting lipid droplet accumulation. Oil Red O staining; scale bar = 50 μm.

The Oil Red O staining of lipid droplets accumulated in free fatty acids (FFAs)-treated HepG2 hepatocytes in the presence of different concentrations of fluid extracts is presented in Figure 2.

Light micrographs showed that fatty-acid-stressed cells treated or not with fluid extracts presented the ballooning morphology characteristic for steatosis, unlike the untreated hepatocytes. In addition, the model presented the accumulation of lipid particles that crossed the cell membrane barrier, simulating the liver uptake of albumin-bound fatty acid released from adipose tissue into the bloodstream [58].

**Figure 3:**
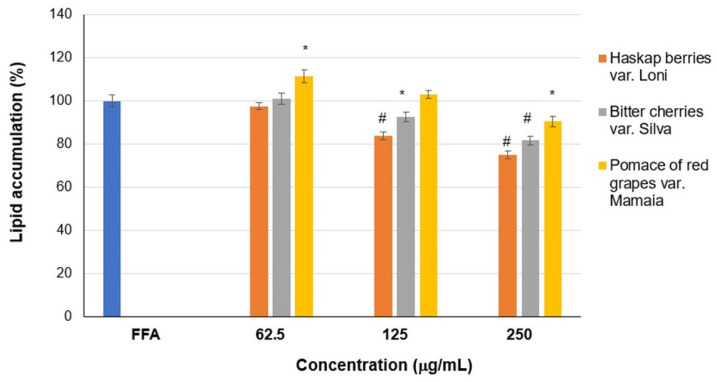
Lipid accumulation in HepG2 cells treated with FFAs and different concentrations of fluid extracts of haskap berries var. Loni, bitter cherries var. Silva, and pomace of red grapes var. Mamaia, determined via Oil Red O extraction. The results are mean ± SD (*n* = 6); * *p* < 0.05 and ^#^ *p* < 0.01, compared to FFAs-treated cells (positive control).

The quantification of extracted Oil Red O revealed that the treatment with fluid extracts of fatty-acid-stressed cells resulted in the significant (*p* < 0.05) inhibition of lipid accumulation (Figure 3). Thus, a lipid decrease between 16 and 25% was observed in the case of haskap berry extract treatment and between 7 and 18% in the case of bitter cherry extract treatment, at 125 and 250 μg/mL concentrations, compared to the lipid accumulation in FFAs-treated hepatocytes (100%, positive control). Similarly, only a 10% decrease was found in the case of treatment with the fluid extract of red grape pomace var. Mamaia at 250 μg/mL.

The lipid lowering mechanisms in an excessive fatty acid milieu are not fully understood. It has recently been debated whether the lipid accumulation in liver cells is a cause or a consequence of hepatocyte-altered metabolism, leading to hepatic steatosis or even the progression of liver disease [59]. However, it has been demonstrated that the interaction between lipid droplets and hepatic cells is driven by major lipid metabolism pathways activated through AMPK and sterol regulatory element-binding transcription factor 1 (SREBP-1) [60]. Previous in vitro studies showed that several types of polyphenols, such as flavonoids, stilbenes, and anthocyanidins, could regulate the mitochondria dysfunction such that a reduction in lipid accumulation was observed [61]. In the case of haskap berry extract, the promotion of AMPK phosphorylation and the decrease in SREBP-1c, PPARγ, and CCAAT-enhancer-binding protein α (C/EBPα) transcription factor expression was previously reported, thus suppressing lipogenesis and presenting anti-obesity potential [15]. This action was confirmed in mice on a high-fat diet supplemented with haskap berry extract via liver histological observations and biochemical determinations at both the gene and protein level [14]. Nutrigenomics showed that liver lipid accumulation and plasma IL-6 were reduced after a diet with standardized sweet cherry (*P. avium* var. Bing) powder depleted of anthocyanin through the modulation of PPARδ mRNA expression in obese diabetic (db/db) mice [62]. A commercial proanthocyanidins extract from grape seeds demonstrated the protection of the liver by reducing lipid droplet formation and triglyceride content, and promoting β-oxidation through the genetic mechanisms of the mRNA expression regulation of SREBP-1c and PPAR-α in rats on a high-fat diet, but cellular fatty acid uptake was not elucidated, as phenolics could exert a pleiotropic effect on the organism [63].

#### 2.3.3. Effect on ROS, Lipid Peroxidation, and Nitric Oxide

**Figure 4:**
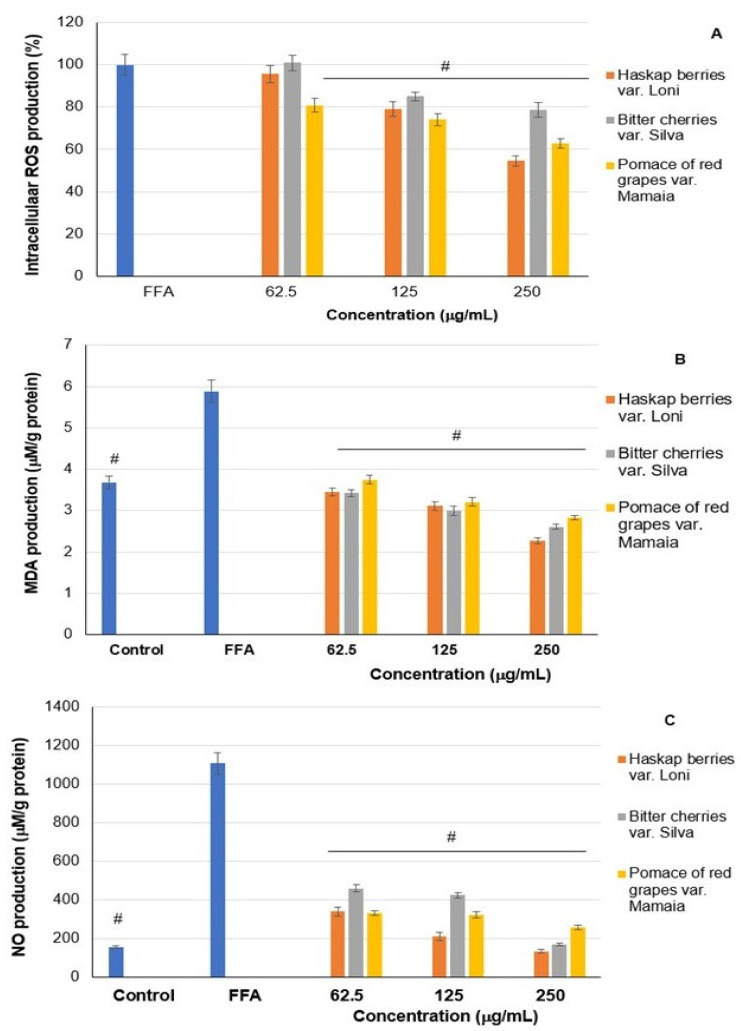
Intracellular ROS (**A**), MDA (**B**), and nitric oxide (NO) production in HepG2 cells treated with FFAs and different concentrations of fluid extracts of haskap berries var. Loni, bitter cherries var. Silva, and pomace of red grapes var. Mamaia, determined using flow cytometry, TBARS, and Griess assay, respectively. The results are mean ± SD (*n* = 6) (**A**) and mean ± SD (*n* = 9) (**B**,**C**); ^#^ *p* < 0.01, compared to FFAs-treated cells (positive control).

The effect of fluid extracts on redox and lipid peroxidation processes was investigated in stressed HepG2 hepatocytes. The results of intracellular ROS, MDA, and nitric oxide production in cells cultivated in the presence of different concentrations of fluid extracts are presented in Figure 4A–C.

The data in Figure 4A showed a dose-dependent decrease in ROS production in cells treated with fluid extracts compared to fatty-acid-treated cells. Thus, ROS production diminished in a higher proportion in the case of treatment with haskap berry extract by 4–45% and pomace of red grapes var. Mamaia extract by 19–37%, and in the case of bitter cherry extract treatment by 15–21%.

Similarly, Figure 4B showed a significant (*p* < 0.05) decrease in MDA concentration in cells treated with fluid extracts at all tested concentrations compared to fatty-acid-treated cells (positive control). Thus, the MDA production decreased in the following descending order of inhibitory capacity: a reduction of 41–61% in the case of haskap berry extract treatment, 42–56% for bitter cherry extract, and 36–52% for pomace of red grape extract.

The results of nitric oxide production in fatty-acid-stressed HepG2 hepatocytes cultivated in the presence of different concentrations of fluid extracts showed a significant (*p* < 0.01) decrease in nitric oxide secretion in cells treated with fluid extracts at all tested concentrations compared to fatty-acid-treated cells (positive control) (Figure 4C). The treatment with haskap berry extract had an inhibitory effect of 70–88% and bitter cherry extract lowered by 58.5–85%, while pomace of red grapes var. Mamaia extract lowered by 30–77% compared to nitric oxide production in fatty-acid-treated cells. Each fluid extract had a dose-dependent effect on reducing the level of nitric oxide. It was observed that the fluid extracts from Romanian cultivars of bitter cherries and grape pomace has similar efficiency as haskap cultivar, decreasing ROS, MDA, and nitric oxide levels.

MDA and nitric oxide are the final products of lipid peroxidation cascade, representing markers of oxidative stress and inflammation, respectively. Previous studies showed that serum and hepatic MDA levels were significantly reduced in mice on a high-fat diet with Korean haskap ethanolic extract [14], Muscat Bailey A grape stalk extract [64], and sour cherry seed powder [23], indicating their capacity to protect against lipid peroxidation and ameliorate liver steatosis. The accumulation of lipids in the liver might trigger inflammation, which together with ROS could lead from steatosis to steatohepatitis [65]. A close relationship was reported between the expression of antioxidant-related genes, like *HO-1*, *Nqo1*, and *Gclc*, regulated by nuclear factor-erythroid 2 related factor 2 (Nrf2), and the level of proinflammatory cytokines that intervene in the production of nitric oxide [12]. A favorable in vitro effect was found in the case of lyophilized Korean and Chinese haskap fruits added in the cultivation medium of oxidative-stressed HepG2 cells and inflamed mouse macrophages, indicating hepatoprotective activity [12]. The effect of an ethanolic extract of sweet cherry (cv. Saco) on nitric oxide level was previously reported in HepG2 cells incubated with sodium nitroprusside due to inducible nitric oxide synthase (iNOS) ligand modulation by phenolic compounds, which was also demonstrated by the docking results [22]. Protection against hepatic injury induced in mice was revealed in the case of *P. cerasus* kernel [66] and black cherry (*P. serotina* Ehrh.) [21] extract administration. An ethanolic extract of red grape pomace injected into Wistar rats could reduce nitrate production in a concentration-dependent rate, showing its antioxidant and anti-inflammatory activity [67]. Hepatoprotection against CCl_4_-induced liver injury in mice was revealed for a polysaccharidic extract of red grape pomace Cabernet variety that regulated the level of oxidative stress and inflammation, acting in vivo on hepatic antioxidant enzymes superoxide dismutase and catalase, and glutathione, but also on the proinflammatory cytokines tumor necrosis factor α (TNF-α) and IL-6 [41]. Moreover, the administration of grape seed oil had a similar hepatoprotective effect in the liver of γ-irradiated rats and anti-inflammatory activity through the down-regulation of cytochrome P450 2E1 (CYP2E1) and iNOS gene expression via NF-kB, besides an antiapoptotic activity mediated by caspase-3 gene expression modulation [40].

## 3. Materials and Methods

### 3.1. Plant Material and Reagents

The genotype Loni of haskap (*L. caerulea* L.) was obtained via the free hybridization of *L. caerulea* L. var. edulis selections and approved in 2003 by ICDP Maracineni, Romania. The genotype Silva of bitter cherry (*P. avium* syn. *Cerasus avium* var. *sylvestris*) was obtained and approved in 1983 by ICDP Maracineni, Romania. The genotype Mamaia of red grapes (*V. vinifera* L.) was obtained through the sexual hybridization of Merlot, Babeasca Neagra, and Muscat Ottonel selections and approved in 1991 by the SCDVV Murfatlar, Romania.

**Figure 5:**
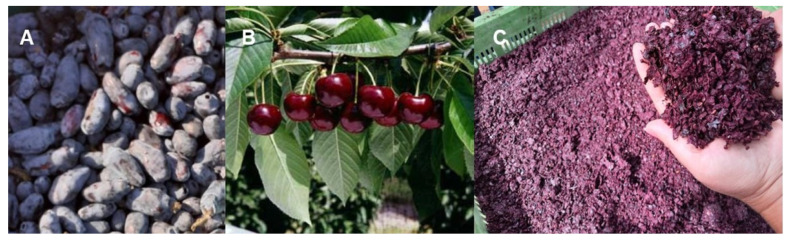
Ripe fruits of Romanian genotypes of haskap (*L. caerulea* L.) var. Loni (**A**), bitter cherry (*P. avium* L. var. *sylvestris*) var. Silva (**B**), and pomace of red grapes (*V. vinifera* L.) var. Mamaia (**C**) (original photos by A.R., T.N.-P., V.A.).

For this study, ripe fruits of haskap var. Loni (Figure 5A) were harvested from 6 shrubs from the Moldova region in April–May 2022. Ripe fruits of bitter cherry var. Silva (Figure 5B) were harvested from 3 trees from the Dobrogea region in July 2019. Pomace (Figure 5C) was obtained from red grapes var. Mamaia harvested from 10 stumps from the Dobrogea region in August 2022 and processed for wine production. The biologic material was transported to the lab in portable refrigerator at 4 °C.

2,2′-azino-bis(3-ethylbenzothiazoline-6-sulfonic acid) diammonium salt (ABTS), DPPH and 2′,7′-dichlorofluorescein diacetate (DCFH-DA), 3-[4,5-dimethylthiazole-2-yl]-2,5-diphenyltetrazolium bromide (MTT) were purchased from Sigma-Aldrich (Darmstadt, Germany). All chemical reagents were of analytical purity and purchased from Sigma-Aldrich (Darmstadt, Germany), unless otherwise specified. HPLC reagents and standards were of HPLC grade and purchased from Merck (Darmstadt, Germany). HepG2 cell line of human hepatocellular carcinoma was from ATCC. Dulbecco’s Modified Eagle Medium (DMEM), fetal bovine serum (FBS), and antibiotic mixture were purchased from Sigma-Aldrich (Darmstadt, Germany).

### 3.2. Preparation of Fluid Extracts

Haskap berries, pitted cherries, and red grape pomace were air-dried in an oven with ventilation (Memmert, Buchenbach, Germany) at 40 °C. Then, they were ground to a fine powder using an electric grinder (Bosch, Gerlingen, Germany). The powder (10 g) was extracted in ethanol/water (70/30, *v*/*v*), in a ratio of 1:10 (*w*/*v*) through ultrasonication using UP200Ht ultrasonic homogenizer (Hielscher Ultrasonics, Teltow, Germany), at 200 W, 26 kHz, at room temperature, for 15 min. The extracts were filtered through Whatman No. 1 filter paper. The solvent was removed in vacuo at a rotary evaporator (Heidolph Instruments, Schwabach, Germany) at 40 °C. The extracts were lyophilized at a Gamma 1–16 LSC freeze dryer (Christ, Osterode am Harz, Germany) and stored in sealed containers in a dry, dark place until the analyses. The extraction yield was calculated as dry weight percentage from the initial dry weight.

### 3.3. Determination of Total Phenolic, Flavonoid, Anthocyanin, and Ascorbic Acid Content

The total phenolic content of the fluid extracts was determined using a slightly modified Folin–Ciocalteu assay [68]. Briefly, a mixture of extract sample (150 μL) and Folin–Ciocalteu reagent (750 μL) was incubated at room temperature for 5 min. Then, a solution of 12% (*w*/*w*) sodium carbonate (2 mL) was added and the volume was made up to 15 mL with distilled water, followed by incubation at room temperature for 30 min. The absorbance (Abs) was measured at a wavelength of 765 nm using a UV/VIS spectrophotometer V650 (Jasco Corporation, Hachioji, Japan). A standard curve was built using six serial dilutions of a stock solution of 500 μg/mL caffeic acid. The results were calculated from the regression equation and were expressed as CAEs.

The total flavonoid content of the fluid extracts was determined using an aluminum chloride assay [69]. Mixtures of extract sample (0.5 mL), methanol (1.5 mL), a solution of 10% aluminum chloride (0.1 mL), 1 M sodium acetate (0.1 mL), and distilled water (2.8 mL) were incubated at room temperature for 30 min. Abs was measured at a wavelength of 415 nm using a UV/VIS spectrophotometer V650 (Jasco Corporation, Hachioji, Japan). A standard curve was built using six serial dilutions of a stock solution of 500 μg/mL quercetin. The results were calculated from the regression equation and were expressed as QEs.

Total anthocyanin content was determined using the pH differential method [70]. Briefly, a volume of sample (1 mL) was diluted in 0.025 M KCl buffer, pH 1, and 0.4 M sodium acetate buffer, pH 4.5, respectively, and vortexed. After 20 min of incubation at room temperature, Abs was read at 520 and 700 nm wavelengths using a UV/VIS spectrophotometer V650 (Jasco Corporation, Hachioji, Japan). The results were calculated from the Abs differences at pH 1 and pH 4.5 and were expressed as CGEs.

Ascorbic acid content was determined using a Folin–Ciocalteu reagent, as previously described [71]. Briefly, a volume of 1 mg/mL sample (1 mL) was thoroughly mixed with Folin–Ciocalteu reagent diluted 1:5 (5 mL) and incubated at room temperature, in the dark, for 3 min. Abs was read at a wavelength of 765 nm using a UV/VIS spectrophotometer V650 (Jasco Corporation, Hachioji, Japan). A standard curve was built using six serial dilutions of a stock solution of 500 μg/mL ascorbic acid.

### 3.4. HPLC Analysis

Fluid extracts were analyzed using HPLC carried out on an Agilent 1200 system (Agilent Technologies Inc., Santa Clara, CA, USA) provided with a diode array detector. The sample (10 μL) was injected and separated on C18 Zorbax XDB reverse phase column (4.6 × 150 mm) thermostatted at 27 °C. The mobile phase consisted of 20 mM phosphoric acid buffer, pH 2.12 (A), and acetonitrile (B) in a gradient of 2–20% B in A, 30 min, 20–30% B in A, 10 min, 30% B in A, 10 min, 30–2% B in A, 10 min, 2% B in A, 15 min. The flow rate was kept at 0.7 mL/min and peaks were detected at 260, 280, and 320 nm wavelengths. The identification of main phenolic acids and flavonoids was carried out according to the retention time and their quantification based on the calibration curves built for each phenolic standard and corresponding regression equations (Table S1) by peak area integration using Chemstation B 03.01 SR1 software. The results were expressed as mg/100 g dry weight.

### 3.5. Determination of Free Radical Scavenging Capacity

TEAC assay is based on the scavenging of the cationic radicals of ABTS. The scavenging capacity of fluid extracts was analyzed according to a previously described protocol [68]. Briefly, a stock solution prepared as a mixture of 7 mM ABTS solution and 2.45 mM potassium persulfate, in a ratio of 1:1 (*v*/*v*) and incubated at room temperature in the dark for 16 h was diluted to reach an Abs of 0.70 ± 0.02. Then, different concentrations of the sample were incubated with ABTS reagent in a ratio of 1:10 (*v*/*v*), at room temperature, in the dark, for 10 min. Abs was measured at a wavelength of 734 nm using a UV/VIS spectrophotometer V650 (Jasco Corporation, Hachioji, Japan). A standard curve was built using six serial dilutions of a stock solution of 250 μM Trolox, a known antioxidant agent. The results were calculated from the regression equation and were expressed as TEs.

The second assay is based on the inhibition of the free radicals of DPPH. The inhibition capacity of fluid extracts was analyzed according to an adapted protocol [72]. Briefly, different concentrations of the sample (150 μL) were incubated with 0.25 mM DPPH solution (1.35 mL) and 0.1 M Tris-HCl buffer, pH 7.4 (0.9 mL), at room temperature, in the dark, for 30 min. Abs was measured at a wavelength of 517 nm using a UV/VIS spectrophotometer V-650 (Jasco Corporation, Hachioji, Japan). A control was similarly processed, replacing the sample with buffer. The inhibition capacity was calculated using the following equation:

Free DPPH radicals inhibition (%) = (Abs_control_ − Abs_sample_)/Abs_control_ × 100 (1)

The inhibitory concentration of 50% free DPPH radicals (IC_50_) was determined from the linear regression equation.

### 3.6. In Vitro Cytotoxicity Testing

#### 3.6.1. Cell Culture and Treatment

Cells from the human hepatoma HepG2 cell line were grown in DMEM supplemented with 10% FBS and 1% antibiotic mixture (100 U/mL penicillin, 100 mg/L streptomycin, 500 mg/L neomycin). Confluent cells were trypsinised and cultivated in the same culture medium, in standard conditions of humidified air with 5% CO_2_, at 37 °C. For the experiments, cells were seeded at a density of 5 × 10^4^ cells/mL in 96-well culture plates and cultivated in standard conditions for 24 h. A stock solution (1 mg/mL) was prepared via the incubation of plant extracts in DMEM, at 37 °C, for 24 h. Then, serial dilutions of stock solution were added over adhered cells and cultivated in standard conditions for 24 h. According to ISO 10993-5 [73], a negative control represented by untreated cells and a positive control represented by cells cultured in the presence of 100 μM H_2_O_2_ were cultivated in the same conditions. Cell viability was assessed using MTT assay, as described below.

#### 3.6.2. MTT Assay

This assay is based on the evaluation of MTT reduction to formazan via the mitochondrial succinate dehydrogenases in viable cells [74]. At the end of incubation, the culture media from each well were removed and a solution of 0.25 mg/mL MTT (100 μL) was added to the cells. The plate was incubated in standard conditions for 3 h, and then isopropanol was added over the cells and gently stirred for 15 min. Abs was measured at a wavelength of 570 nm using a Sunrise microplate reader (Tecan Austria GmbH, Grodig, Austria). The cell viability was expressed as a percentage from untreated cells, which were considered 100% viable.

### 3.7. In Vitro Experimental Model of Steatosis

#### 3.7.1. Cell Culture and Treatment

The hepatoprotective activity was evaluated using an in vitro experimental model of liver steatosis, as previously described [14], with modifications. Adhered HepG2 cells were cultivated in DMEM supplemented with 1% bovine serum albumin and a 0.6 mM mixture of oleic and palmitic acids (1:1) in standard conditions for 24 h. The induction of steatosis was carried out in the presence of samples (62.5, 125, 250 μg/mL) or in their absence (positive control). Cells cultivated in DMEM supplemented with 1% bovine serum albumin served as the negative control.

#### 3.7.2. Determination of Intracellular Lipid Accumulation through Oil Red O Staining

HepG2 cells from the steatosis experimental model cultivated with or without plant extracts were washed in phosphate-buffered saline, pH 7.4, and fixed in 10% formalin for 1 h [75]. Then, cells were stained with Oil Red O solution at room temperature for 30 min and, finally, washed. For cell morphology observations, the cultures were visualized using an inverted microscope Axio Observer D1 (Zeiss Group, Oberkochen, Germany). The content of Oil Red O was determined by adding isopropanol to each well and incubating the plate at room temperature for 10 min with gentle stirring. Abs was measured at a wavelength of 500 nm using a Sunrise microplate reader (Tecan Austria GmbH, Grodig, Austria).

#### 3.7.3. Determination of Intracellular ROS Production Using Flow Cytometry

In the same experimental model of adhered HepG2 cells treated with fatty acids, in the presence or absence of samples, the intracellular ROS production was evaluated using the cell permeant fluorogenic dye DCFH-DA [74]. Briefly, cells cultivated as described in Section 3.7.1. were incubated with 10 μM DCFH-DA at room temperature in the dark for 30 min. Then, cells were analyzed at the end of the treatment using a BD LSR II flow cytometer (Becton Dickinson, Franklin Lakes, NJ, USA). The fluorescence intensity was calculated as a percentage from FFAs-treated cells, considered 100%, using FACSDiva and FlowJo software, and was proportional to ROS production.

#### 3.7.4. Determination of Lipid Peroxidation Using TBARSs Assay

The stressed cells were lysed, centrifuged at 10,000× *g* at 4 °C for 10 min, and the protein content was determined using a bicinchoninic acid kit. The supernatant served to evaluate MDA production, according to a previously described protocol [76]. Briefly, a mixture of 1 mL thiobarbituric acid reagent and 0.5 mL supernatant was incubated in acid medium at 95 °C for 15 min. Abs was measured at a wavelength of 532 nm using a UV/VIS spectrophotometer V650 (Jasco Corporation, Hachioji, Japan). The results were calculated as µM MDA/g protein from the equation of the Beer–Lambert law, considering that *ɛ* was 1.55 × 10^6^ M^−1^cm^−1^.

#### 3.7.5. Determination of Nitric Oxide Using a Griess Assay

The nitrite concentration was determined based on the diazotization reaction using a Griess Reagent System kit (Promega, Madison, WI, USA), according to the manufacturer’s instructions. Briefly, mixtures of sulfanilamide solution and supernatant in a ratio of 1:1 (*v*/*v*) were incubated in the wells of a 96-well culture plate in the dark for 10 min. Then, 50 μL N-1-naphthylethylenediamine dihydrochloride solution was added to each well and the incubation continued for 10 min. Abs was read at a wavelength of 550 nm using a Spectrostar Nano microplate reader (BMG Labtech, Ortenberg, Germany). A standard curve was built using eight serial dilutions of 100 μM nitrite stock solution, a primary breakdown product of nitric oxide. The results were calculated from the regression equation and were expressed as µM/g protein.

### 3.8. Statistical Analysis

Experiments were carried out as three repetitions in duplicate (*n* = 6) or in triplicate (*n* = 9). The results were expressed as mean ± SD. Statistical analysis on pairs of interest was performed with a two-tailed, two-sample equal variance Student *t*-test. Significant differences were considered at *p* < 0.05. Pearson correlation coefficients (r^2^) were calculated using Microsoft Office software (2019).

## 4. Conclusions

The obtained results revealed the complex composition of the fluid extracts prepared from the Romanian cultivars of haskap berries (*L. caerulea* L.) var. Loni, bitter cherries (*P. avium* L. var. *sylvestris* Ser.) var. Silva, and the pomace of red grapes (*V. vinifera* L.) var. Mamaia and the correlation with their antioxidant activity. We confirmed the hepatoprotective activity of the fluid extract of haskap berry cultivar and demonstrated, for the first time, the similar properties of bitter cherry and red grape pomace cultivar extracts that were also able to protect human hepatocytes in an in vitro experimental model of steatosis. This study clearly showed the capacity of all vegetal fluid extracts to inhibit lipid accumulation induced by fatty acids in HepG2 cells, modulate the redox processes via the intracellular ROS production, inhibition, and interruption of lipid peroxidation cascade reactions and the formation of MDA, and decrease the level of nitric oxide. Consequently, this indicated that the analyzed fluid extracts were good lipid overload mediators and had antioxidant properties for further exploitation as promising nutraceuticals for NAFLD prevention and treatment. This study could serve as a basis for future research that will define novel nutraceutical formulations from vegetal extract mixtures with synergistic action in ameliorating hepatic steatosis and obesity in order to deepen our understanding of the molecular mechanisms of lipid metabolic dysfunction.

## Figures and Tables

**Table 1:** Total phenolic, flavonoid, anthocyanin, and ascorbic acid content of fluid extracts of Romanian cultivars of haskap berries var. Loni, bitter cherries var. Silva, and pomace of red grapes var. Mamaia. The results are expressed as mean ± standard deviation (SD) (*n* = 9).

Fluid Extract	Total Phenolic Content * (mg CAEs/g Dry Weight)	Total Flavonoid Content * (mg QEs/g Dry Weight)	Total Anthocyanin Content* (mg CGEs/g Dry Weight)	Ascorbic Acid Content (mg/g Dry Weight)
Haskap berries var. Loni	75.21 ± 2.44	5.53 ± 0.60	0.41 ± 0.02	21.07 ± 1.85
Bitter cherries var. Silva	4.43 ± 0.21	0.35 ± 0.02	0.16 ± 0.01	2.54 ± 0.16
Pomace of red grapes var. Mamaia	247.29 ± 9.16	17.16 ± 0.37	0.39 ± 0.02	15.27 ± 1.15

* Total phenolic content expressed as caffeic acid equivalents (CAEs); total flavonoid content expressed as quercetin equivalents (QEs); total anthocyanin content expressed as cyanidin-3-glucoside equivalents (CGEs).

**Table 2:** HPLC analysis of phenolic acids and flavonoids quantified in fluid extracts of haskap berries var. Loni, bitter cherries var. Silva, and red grape pomace var. Mamaia. The results are expressed as mean ± SD (*n* = 6).

Compound	Quantity (mg/100 g Dry Weight)		
	Haskap Berries var. Loni	Bitter Cherries var. Silva	Pomace of Red Grapes var. Mamaia
Chlorogenic acid	126.53 ± 5.37	63.27 ± 2.73	N.D.
Catechin hydrate	134.01 ± 7.79	N.D.	398.49 ± 28.57
Caffeic acid	N.D.	25.24 ± 1.60	40.26 ± 3.08
Syringic acid	461.73 ± 21.51	15.76 ± 0.98	79.96 ± 5.12
Rutin trihydrate	7.16 ± 0.92	N.D.	N.D.
Ferulic acid	9.24 ± 0.86	N.D.	N.D.
Apigenin 7-glucoside	24.64 ± 1.04	28.64 ± 2.44	39.95 ± 2.39
Quercetin 3-β-glucoside	61.09 ± 4.95	N.D.	169.12 ± 8.82
Kaempferol 3-β-glucoside	61.06 ± 3.22	16.30 ± 2.72	128.28 ± 6.15
Myricetin	2.83 ± 0.33	N.D.	32.99 ± 1.71
Rosmarinic acid	14.64 ± 2.80	N.D.	70.47 ± 3.81
Quercetin dihydrate	7.21 ± 2.15	13.88 ± 1.46	23.84 ± 2.26
Apigenin	N.D.	N.D.	N.D.
Kaempferol	N.D.	N.D.	57.78 ± 4.34

N.D.—not determined.

**Table 3:** The free radical scavenging capacity of fluid extracts of haskap berries var. Loni, bitter cherries var. Silva, and pomace of red grapes var. Mamaia determined using TEAC and DPPH assays. The concentration that inhibited 50% of free radicals (IC_50_) was calculated. The results are expressed as mean ± SD (*n* = 6).

Fluid Extract	TEAC * (mM TEs/g Dry Weight)	DPPH Inhibition * (%)	IC_50_ (μg/mL)
Haskap berries var. Loni	1.70 ± 0.11	45.07 ± 3.17	1310.88 ± 78.39
Bitter cherries var. Silva	0.64 ± 0.02	19.86 ± 0.84	3308.82 ± 168.06
Pomace of red grapes var. Mamaia	1.88 ± 0.15	52.25 ± 2.22	922.97 ± 52.18

* TEAC expressed as Trolox equivalents (TEs); % DPPH inhibition at a concentration of 1000 μg/mL fluid extract.

**Table 4:** The Pearson correlation coefficients calculated as free radical scavenging capacity of fluid extracts of haskap berries var. Loni, bitter cherries var. Silva, and pomace of red grapes var. Mamaia vs. total phenolic, total flavonoid, total anthocyanin, and total ascorbic acid content.

	TEAC	DPPH Inhibition
Total phenolic content	0.6575	0.7292
Total flavonoid content	0.6748	0.7453
Total anthocyanin content	0.9579	0.9211
Total ascorbic acid content	0.8142	0.7500

Strong correlation, >0.8; medium correlation, 0.5–0.8.

## Data Availability

The data presented in this study are available on request from the corresponding author.

## Supplementary Materials

The supporting information can be downloaded at: https://www.mdpi.com/article/10.3390/ijms242316849/s1.
