# Supplementary material for: The Chemical Profile, Antioxidant, and Anti-Lipid Droplet Activity of Fluid Extracts from Romanian Cultivars of Haskap Berries, Bitter Cherries, and Red Grape Pomace for the Management of Liver Steatosis
Source: Int J Mol Sci. 2023 Nov 28;24(23):16849. doi: 10.3390/ijms242316849 (PMC10706173; doi:10.3390/ijms242316849)
Supplement: Supplementary file 1 [file ijms-24-16849-s001.zip › ijms-2693843-supplementary.pdf]

Table S1. Regression equation and correlation coefficient of the calibration curves of standards used in quantitative analysis of fluid extracts by HPLC

**Compound**	**Retention time (min)**	**Wavelength (nm)**	**Regression equation**	**Correlation coefficient (r^2)**
Chlorogenic acid	19.694	320	y= 35.54x-18.08	r^2 = 0.9972
Catechin hydrate	20.385	280	y= 8.26x-21.67	r^2 = 0.9990
Caffeic acid	21.550	320	y= 70.53x-92.95	r^2 = 0.9970
Syringic acid	22.190	280	y= 73.56x-61.87	r^2 = 0.9986
Rutin trihydrate	30.167	320	y= 53.66x-47.00	r^2 = 0.9979
Ferulic acid	31.177	320	y= 17.81x-26.46	r^2 = 0.9984
Apigenin 7-glucoside	31.586	320	y= 44.76x-62.67	r^2 = 0.9982
Quercetin 3-β-glucoside	32.753	320	y= 9.08x=18.27	r^2 = 0.9822
Kaempferol 3-β-glucoside	35.936	320	y= 18.69x-6.04	r^2 = 0.9978
Myricetin	38.400	320	y= 50.37x-17.89	r^2 = 0.9981
Rosmarinic acid	39.407	320	y= 12.38x-30.85	r^2 = 0.9974
Quercetin dihydrate	43.909	260	y= 53.25x-29.32	r^2 = 0.9980
Apigenin	49.434	320	y= 67.13x-45.60	r^2 = 0.9982
Kaempferol	51.356	260	y=47.93x-22.36	r^2 = 0.9973
